# A network perspective of human–nature interactions in dynamic and fast-changing landscapes

**DOI:** 10.1093/nsr/nwad019

**Published:** 2023-01-17

**Authors:** Örjan Bodin, Haibin Chen

**Affiliations:** Stockholm Resilience Centre, Stockholm University, Stockholm 106 91, Sweden; College of Economics and Management, Northwest Agriculture and Forestry University, Yangling 712100, China

**Keywords:** social-ecological system, collaborative governance, institutional fit, changing landscapes, adaptation, networks

## Abstract

Increasing and intensifying the use of land represents a prominent sustainability challenge of particular importance in regions undergoing rapid change while at the same time exhibiting large natural and anthropocentrically induced variability. To reconcile the needs for both human prosperity and healthy ecosystems, a more integrated understanding of key biophysical and adaptation processes is paramount in such dynamic and deeply entangled social and environmental contexts. Interdisciplinary research utilizing a network perspective provides a novel methodological and theoretical approach to that end. We review and synthesize recent network-centric studies, and use this network perspective to show how rangeland managers in a dynamic pastoral region in the Qinghai Province of China form social relationships based on geographic proximity, social status and shared grazing areas. The results indicate that adaption to biophysical and socioeconomic changes is partly a social process in that rangeland managers develop their adaptive capacity jointly and in concert with others they trust and with whom they share grazing areas. Avenues for further development of this network perspective, in terms of how it might contribute important new insights about how to sustainably use land in dynamic landscapes undergoing rapid change, are suggested.

## Teaser

Reviewing and synthesizing how an interdisciplinary network perspective can inform on governance and adaptation processes in regions undergoing rapid social-, economical-, and environmental changes.

## INTRODUCTION

**Figure 1.**
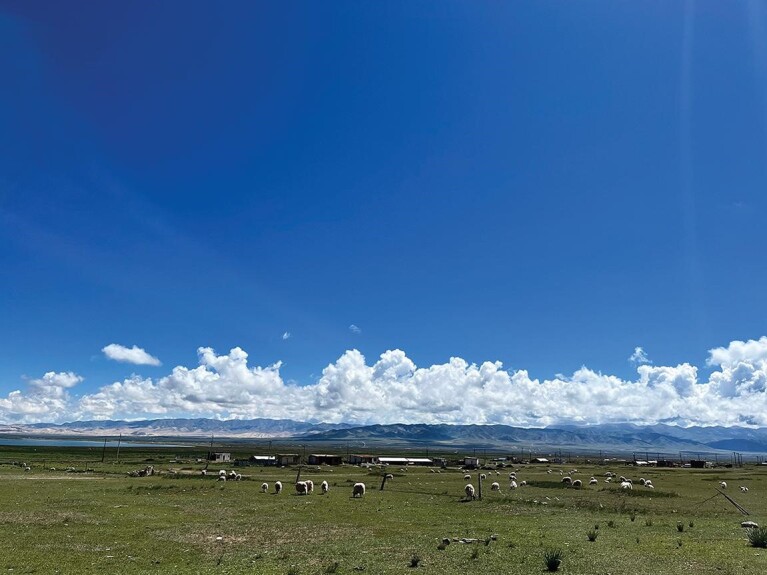
A rangeland in the Qinghai-Tibetan Plateau. The grassy fields are mainly used as winter grazing areas for sheep and yak, whereas the summer grazing areas are typically located in the mountainous areas in the background. Photo credit: Shan Jiang.

In the Anthropocene, human enterprise has been driving drastic changes in the Earth's biosphere and has caused multiple environmental challenges, such as climate change, biodiversity degradation, pollution and habitat destruction [1]. Landscapes are the principal source of a multitude of services the ecosystems provide that benefit human well-being, yet are subject to the most prominent modification by humans [2]. Sustaining a flow of multiple ecosystem services from landscapes thus constitutes a grand challenge, which is particularly acute in regions undergoing rapid economic and population growth while being characterized with large natural and anthropocentrically induced variability [3]. This challenge is prominent in China and other Asian countries, as well as on the African and South American continents. The Qinghai-Tibetan Plateau (QTP), for instance, a key area of biodiversity and freshwater supply for China, has ∼165 million hectares or 42% of China's rangeland and provides livelihoods for over 6 million pastoralists and agro-pastoralists, a majority of which belong to an ethnic minority [4]. At this so-called ‘Third Pole of the Earth’, the living environment is extremely harsh, characterized by hypoxia, frigidity, aridity and significant variability of resource availability (Fig. 1). Climate change has been putting further strain on the viability of herder livelihoods by inducing more frequent, intense shocks from extreme weather events such as droughts and snow storms [5]. Moreover, national efforts to integrate local pastoral communities into broader markets have led to a rapid succession of institutional changes, e.g. the consecutive rollout of the Household Contract Responsibility System [6], the Payment for Ecosystem Services Programs [7] and the Herdsman Settlement Initiatives [8]. Given these changes, it is clear that smallholders in this vast and variable landscape need to constantly adapt to varying social- and biophysical conditions in order to maintain their livelihoods [9]. Strikingly, some locally adapted innovations, such as the community-based grazing quota system, which embeds market mechanisms into customary institutions and leverages the traditional social norms of trust and reciprocity, kinship networks, and local ecological knowledge, have shown a remarkable capacity to enhance communities’ abilities to respond to these challenges [8,10].

Another prominent example of a region undergoing rapid change is the Sahel region of Africa, a belt across the African continent characterized by a tropical semi-arid climate located south of the Sahara Desert and north of the Sudanian Savanna. The region constitutes a very challenging biophysical environment, typified by low and highly variable rainfall, and increasingly suffers from climate change and increased population pressure, as 50%–72% of the inhabitants depend mainly on farming and pastoralism for their livelihoods [11]. Nonetheless, Nigerian farmers have demonstrated that these rather harsh conditions are not impossible to overcome. By enhancing the tree cover, their capacity to withstand droughts and sustain their livelihoods has increased [12]. Within regions in Burkina Faso, the agricultural yields have, in spite of these challenges, increased in parallel with a large population growth. Furthermore, by deliberately interspersing croplands with trees and scrubs, the negative impacts of increased croplands on ecosystem services stemming from woody vegetation have been kept moderate [9].

In sum, these examples demonstrate that in rapidly changing and intrinsically variable landscapes, governance arrangements that can promote a high degree of adaptability and resilience to internal and external changes and disturbances are key [13,14]. Of particular relevance in these contexts are the strong and direct interdependencies between the people living in these landscapes, and the constantly changing biophysical conditions. Accordingly, a deeper scientific understanding of the complexities of dynamic and intertwined social-ecological systems (SESs) is commonly called for [1,2,15,16]. To this end, interdisciplinary research approaches that incorporate different perspectives, insights and methods are paramount for a more comprehensive study of biophysical and governance processes in tandem [17,18].

### A social-ecological perspective on dynamic landscapes

Governing dynamic landscapes is challenging because of the multiplicity of stakeholders, the sociopolitical and biophysical permeability of human-demarcated political and administrative jurisdictions and the within- and cross-scale dynamics of biophysical processes that are characterized by known and unknown uncertainties [16,19]. These complex social and biophysical entanglements imply that no single actor nor stakeholder are able to solely address all governance challenges. Thus, land use governance is, in part, favourably understood as a collective action problem emphasizing the need for a multitude of actors to find ways to jointly address common challenges [20,21]. Collective action problems are often difficult to address, but they are not insurmountable. Indeed, a large and growing body of empirical evidence, especially relating to management of common pool resource (CPR), has pointed to the necessity of organizing stakeholders to devise and implement solutions that align individual incentives with the common good [22]. Collaborative approaches to governance fall into this realm, i.e. governance approaches and frameworks particularly emphasizing the importance of collaboration (e.g. collaborative governance, adaptive governance and adaptive co-management) [23–25]. The term ‘collaborative environmental governance’ is used here to emphasize the importance of collaboration among a multiplicity of actors (landscape managers, government bodies, conservation practitioners, NGOs, research institutes, etc.) at multiple organizational levels to better respond to complex environmental challenges [23,24,26] in a general and inclusive sense without being tightly or exclusively linked to any specific framework. Taken together, collaboration is put forward as a means to (i) promote knowledge integration, innovation and diffusion [27,28]; (ii) solve conflicts and collective dilemmas via coordination and cooperation [29]; (iii) actively adapt to emerging problems from broader spatiotemporal contexts [30].

Governance systems, however, often consist of groups of actors that hold different values, knowledge, interests and power. Reconciling these differences presents a challenge by itself, thus in reality multi-actor collaborative governance initiatives often face problems such as the obstruction and co-optation by vested interest groups [31], the lack of transparency and fairness in regards to decision-making processes [32], and recurring issues of legitimacy and accountability [33]. Unless rival actors engage in sincere conversations, these differences could stall any attempts to collaboratively address pressing environmental and social problems [34,35], while it is acknowledged that discussions alone provide no guarantee that progress can ever be made [36,37].

Weaving collaborative networks alone is not enough to address pressing issues of sustainability in a landscape context. Complexity and uncertainty are two essential features of ecosystems, particularly so in dynamic landscapes [38]. Interdependencies between different ecosystem components are ubiquitous across spatial and temporal scales. Meanwhile, ecosystems are also characterized by uncertainty and emergent properties [39]. Hence, as stated, over the last decades, numerous scholars have highlighted the need to apply a more integrated perspective on environmental governance by factoring in if and how the characteristics of the governed ecosystems effect and are affected by governance processes and structures. In short, if people, organizations and societies are not conceived and studied as embedded in and part of the biophysical realm, we will not be able to more fully understand issues of sustainability [15,40]. Therefore, the research focus should be shifted to the theoretical and empirical investigation of the composition, structure, evolution and processes of collaborative governance systems as situated *within* the biosphere, and we should seek a better understanding of how these social and ecological entanglements shape societies and actors’ abilities to govern integrated SESs more sustainably. In changing landscapes, the intrinsic and direct couplings between people and nature, which are more prominent in intensively used landscapes than in many other contexts, imply that the notion of regarding humans as being decoupled from the natural environment and acting solely within the realm of human societies is hardly justifiable [38,41].

### Towards better institutional fit

Embracing an integrated human-in-nature (or SES) perspective does not, however, deliver any new substantive insights by itself. It merely emphasizes an interdisciplinary and more holistic ambition. The literature on institutional (or social-ecological) fit from the last two decades, however, lends an intuitively appealing lens that helps to guide development towards more substantive insights. Despite the lack of a commonly agreed upon definition of what constitutes institutional fit, the core idea is that effective environmental governance depends in part on an appropriate spatial and temporal alignment of institutional arrangements and sociopolitical processes with the underlying characteristics of the ecosystems being governed [42,43]. Again, this appears as particularly relevant in dynamic landscapes where people and nature are deeply entangled.

Further, the idea of actors being embedded in an environment characterized by uncertainties and interdependencies is not limited to studies of institutional alignments (or fit). By acknowledging the existence of multiple and interdependent action situations or concurrent policy ‘games’, the polycentric governance literature has indirectly investigated the relationship between collaborative approaches to governance and the fragmented but entangled nature of the systems being governed [44]. However, studies that explicitly quantify, assess and explain whether and how governing actors respond to different interdependencies, be they ecological, as being prominent in landscapes, or more related to, for example, overlapping policy subsystems in complex governance settings, are still scarce. Despite a few recent advances [45–48], empirically and quantitatively oriented studies analysing alignments between the characteristics of the governing system and the system being governed remain limited. In particular, due to the difficulties in precisely defining core aspects of institutional fit, the literature typically suffers from idiosyncratic and case-specific operationalizations of institutional fit, making it difficult to generalize or upscale the findings. This, we argue, is limiting our ability to utilize the concept of institutional fit to better understand pressing issues of sustainability in fast-changing landscapes.

### A network perspective on institutional fit

**Figure 2.**
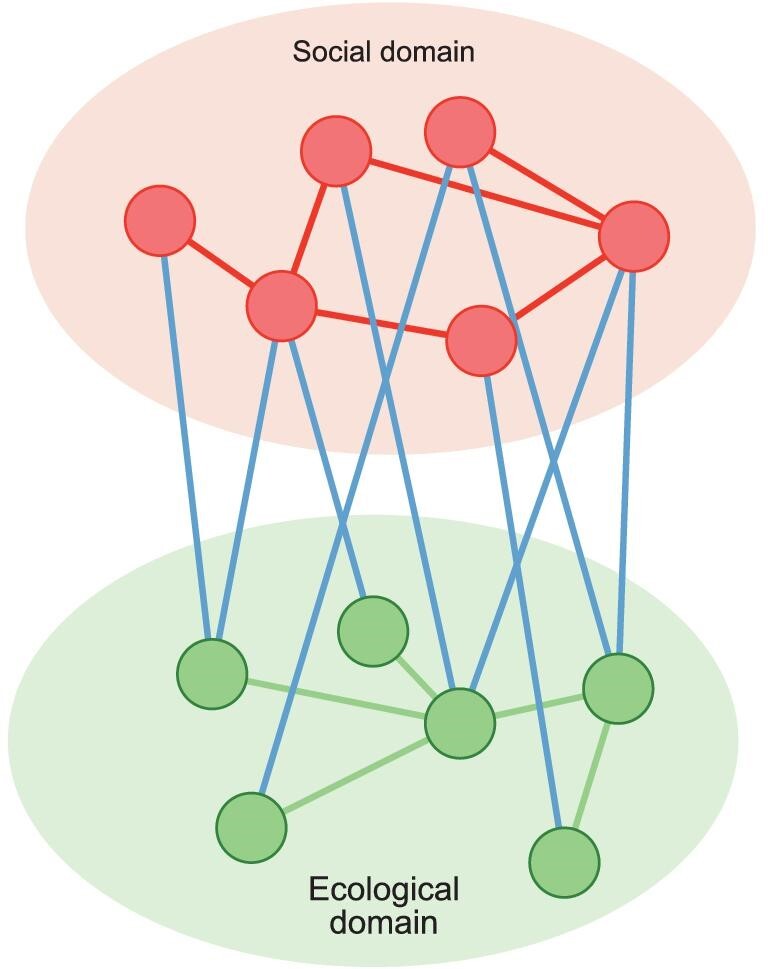
A social-ecological network. The upper ellipse represents the social domain of a social-ecological system, and the lower ellipse the ecological domain. The upper (red) nodes represent actors and the lower (green) nodes represent ecological components. The upper (red) links represent social relationships, such as collaboration. The lower (green) links represent ecological interdependencies, for example species dispersals between habitat patches. The (blue) links connecting the actors and ecological components represent social-ecological interdependencies, such as resource extraction.

Fortunately, recent theoretical and methodological developments in integrated social-ecological-network (SEN) research have been shown to be rather useful and promising with regard to furthering the research on institutional fit. In the same vein, the burgeoning field of network science, which explicitly models and analyses complex systems consisting of numerous parts and their relationships, coincides with these developments [49]. Many studies within sustainability science have applied network concepts and methods to advance research frontiers. Examples include, but are not limited to, interactions of the United Nations Sustainable Development Goals (SDGs) in Agenda 2030 [50], ecosystem services [51] and telecoupling [52]. By representing system components as nodes and their interactions as linkages, network methods clearly embody the entangled nature of SESs, and have a significant strength in analysing the relationships between structures, processes and functions of complex environmental governance systems (Fig. 2).

In particular, recent methodological advances in multilevel exponential random graph models (ERGMs) [53] have been extended to build and model SENs and provide an intriguing tool for addressing questions related to institutional (social-ecological) fit [54]. By defining a set of small network configurations based on theoretically informed hypotheses, the ERGMs have been used to examine and expound the causality between micro-level processes and global observed network structures. For a more detailed review on the foundational developments of SEN analysis for sustainability science, see e.g. [55,56].

This review contributes to this budding research area by reviewing and synthesizing the so-far-rather-limited set of studies that explicitly use network concepts, tools or methods to address environmental governance challenges in dynamic and fast-changing landscapes. We focus on three research topics of SEN literature: the formation, composition and structure of collaborative networks in SESs, aspects of social-ecological fit, and if and how a SEN perspective contributes to our understanding of how actors adapt in fast-changing landscapes. Since there are few network-centric studies addressing dynamic landscapes explicitly, we also draw from network-oriented studies conducted in other contexts. From an institutional fit perspective, we try to hone in on the key findings, key challenges and opportunities when applying a network-centric research approach, with the aim of more fully understanding the governance challenges and of helping to devise more effective governance solutions. To move beyond solely reviewing and synthesizing the current and rather limited literature, a case study about the governance system in a dynamic pastoral region in China will be presented. The objective with the case study is to complement the existing literature and illustrate if and how the SEN perspective can be useful in addressing important research questions related to environmental challenges facing dynamic landscapes across the world.

## A NETWORK-CENTRIC PERSPECTIVE ON GOVERNING DYNAMIC AND FAST-CHANGING LANDSCAPES

Collaborative environmental governance implies that actors and stakeholders are finding ways to collaborate in order to address collective action problems together. These collaborative endeavours can and should entail a wide range of different collaborative processes such as communication and consensus building (see e.g. [57]). Here we direct attention to three important and partly overlapping collaborative processes, i.e. learning, coordination and cooperation. To address complexity and uncertainty in SESs, the literature on environmental governance generally emphasizes the importance of learning, and a consistent argument in favour of collaboration is that by bringing together diverse actors with varied educational backgrounds, occupations, experience and knowledge systems, knowledge integration and innovation can be facilitated [58,59]. Coordination refers to the orchestration of a series of activities among multiple actors with aligned objectives, such as coordinating the actions of land managers to mitigate wildfire risks [60]. In contrast, cooperation usually involves a competitive allocation of scarce resources or public goods, where negotiations are often needed as actors have to reach a compromise, and collective choice rules (e.g. reward and punishment) ought to be in place to provide incentives to comply [21].

While solving collective action problems via collaboration implies improvements at the group level, it is individual landscape managers or organizations who are making decisions to participate in the collaborative endeavours or not. From a rational choice perspective, when actors expect that the benefits from collaboration will be greater than incurred transaction costs (including costs arising from negotiating distribution of benefits/costs, monitoring and rule enforcement), they are more likely to participate [61]. Many extant theories and frameworks on governance and CPR management are, however, inclined to abandon the assumption of strictly rational choice, and instead argue that individuals are boundedly rational and often rely on various heuristics to deal with uncertain and dynamic environments, and decisions are made on the basis of, for example, limited information, subjective beliefs, old habits and imitation of others [62].

In summarizing the above, it becomes clear that the question of whether, how and with what purposes actors choose to engage in collaborative environmental governance is in itself far from a trivial one. This is a research area where a network perspective can help shed light on many of these puzzling questions. From the point of view of participating and potentially collaborating actors, the network perspective directs attention to questions of whether and how various environmental problems have stimulated the formation of collaborative networks: who the collaborators are, with whom and how they collaborate, and how the structures of such collaborative networks relate to the actors’ abilities to address different environmental problems. In this way, it provides an integrated analytical vehicle for investigating the drivers and the performance of collaboration that follows a bottom-up and self-organized approach, as well as for investigating if and how actors engage in collaborative endeavours that are imposed by public authorities in a top-down fashion (e.g. [63]).

### Weaving collaborative social networks

There is a rich literature developing theories and insights on how an actor would engage in a collaboration with other actors. Here, three broadly defined factors are outlined, which have a clear theoretical and methodological link to the perspective of networks. The first factor relates to resource access. Actors with access to important resources like political authority, information, knowledge, technologies and finance are often sought after by others who lack some of these resources [64–66]. Further, these actors with access to critical resources might consequently attain many social relationships in the collaborative network, which also suggests they would therefore possess a greater ability to coordinate actions and influence outcomes [67].

The second factor relates to homophily in a broad sense. In other words, actors who are similar in some sense tend to develop a collaborative relationship with each other (cf. [68]). Similarity among actors often facilitates repeated interactions, which can build trust and reciprocity and in turn further strengthen the relationships over time. Cohesive subgroups (or clusters, communities) are thus likely to form from such processes of reciprocity and transitivity (i.e. two actors who have a relationship with a common third actor also tend to form a relationship with each other). Exactly what type of similarity matters varies between cases and contexts, and could entail factors such as geographic proximity, kinship, occupation, gender, interests and preference [30,65,69].

The third factor is external to the actors, and is instead related to the nature of the collective action problem (cf. [70]). Naturally, there is not just one such external factor, although we here focus on one that is of particular importance in collaborative environmental governance, and that has been coined the ‘risk hypothesis’ [71]. The risk hypothesis stipulates that actors follow different patterns in forming collaborative relationships, depending on if the collective action problem is characterized by high versus low risk. A high-risk problem implies that actors who submit to collaboration would risk suffering significant losses if others choose not to engage in the collaborative process, whereas a low-risk problem implies that collaborating actors would not face such losses if some of the others chose not to engage. These two types of collective action problems align with the distinction between coordination and cooperation, where the former would be associated with low risk and the latter with high risk [54,71]. Further, the risk hypothesis suggests that when actors engage in high-risk collective action problems, they tend to form dense substructures of relationships characterized by reciprocity and transitivity [71]. Dense substructures are argued to be more conducive for monitoring and sanction (i.e. ‘social control’), which is more important in high-risk than in low-risk settings. A low-risk problem would, in contrast, favour less dense network substructures. The risk hypothesis has stimulated a lot of scholarly interest, and some of these original assumptions have been moderated and extended, taking other contextual issues into account such as the overall level of trust within a community of actors, and in what ways actors are connected to the local natural environment [72–74].

### Institutional fit in multilevel social-ecological networks

Many, if not most, environmental problems occurring in landscapes entail social and ecological interdependencies of various sorts, implying that the distribution of the benefits and costs related to actors’ land use decisions often extend across the landscape and the communities of actors. Two general types of interdependencies are commonly highlighted. First, *environmental interdependencies* arise naturally since ecosystems consist of a multitude of biotic and abiotic components that interact across space and time through various types of biophysical processes [75]. This presents a big challenge to environmental governance since societies are typically organized around geographical, jurisdictional and administrative boundaries that are not well aligned with these biophysical processes [76]. Such misalignments are illustrated by the negative impacts of upstream effluent emissions on downstream areas, a lack of action in mitigating invasive plant species range expansion caused by livestock grazers giving rise to spill-over effects to recreational users [77], and reforestation efforts with distal effects across national borders [78].

Second, *policy issue interdependencies* occur when actions stipulated by one policy to address certain environmental problem(s) can entail reinforcing or counteracting consequences for other policies addressing other (or the same) environmental problems [79,80]. For example, a water pollution abatement policy may involve devising rules against ditching since fewer ditches can decrease agricultural runoffs, while at the same time, reduced ditching would also contribute to preventing the loss of wetlands, which in itself constitutes another environmental problem with its own associated policies [81]. Another topic that has spurred substantial scholarly interest in policy issue interdependency are the SDGs that are, as strongly emphasized in Agenda 2030, highly interdependent [82]. These examples illustrate that although policy issue interdependencies can and often do arise due to underlying biophysical interdependencies, they can also arise from how systems of governance and policy developments are constructed from a strictly societal perspective.

**Figure 3.**
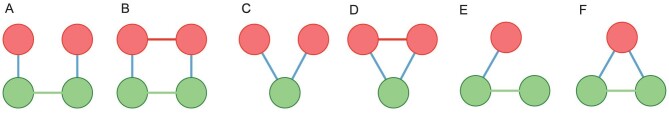
Some foundational social-ecological building blocks. Red nodes represent actors and green nodes represent ecological components. A–D captures different aspects of horizontal institutional (social-ecological) fit and E–F different aspects of vertical institutional fit.

From an institutional fit perspective, the configuration of a collaborative network for environmental governance, discussed in the previous subsection, should thus ideally be well aligned with the interdependencies connecting the ecological components of the governed ecosystems with each other, and with the actors [83] (Fig. 2). The same reasoning applies to policy issue interdependencies within the policy spheres. Exactly what such alignments entail are largely a subject for ongoing research, and here we will focus on some foundational aspects of social-ecological alignment (we refer to it as social-ecological alignment, or ‘fit’, although it also captures the meaning of policy issue and actor alignment). We do so by focusing attention on certain meso-level SEN structures or configurations, which are often called social-ecological building blocks (Fig. 3). A social-ecological building block represents the smallest possible network configuration that nonetheless captures a specific pattern of social-ecological interdependencies that is theorized to come with some important governance implications. Although such building blocks represent stylized and simplistic configurations, while real-world SENs typically are much larger and more structurally complex, they can nonetheless be at the core when analysing real-world SENs. By using statistical analyses building on ERGMs (see e.g. [84]), multilevel SENs can be analysed in a conceptually similar way to ordinary least squares (OLS) regression analyses [85] (see also, Supplementary Data). In doing so, the researchers would select a set of social-ecological building blocks that would then serve as independent variables, while the SEN as a whole constitutes the dependent variable. The estimated coefficients, one per social-ecological building block, would then indicate whether the particular configuration was over- or underrepresented in the network [86]. It is however important to emphasize that the similarity between ERGMs and OLS is only conceptual, since the underlying analytical models are quite different [84].

In elaborating social-ecological alignment and institutional fit, one can distinguish between horizontal and vertical alignment (fit) [54]. Horizontal fit focuses attention towards interdependencies between ecological components (or towards the integrity of the components themselves). These interdependencies represent biophysical processes that are often fundamental to upholding desired functions of ecosystems. For example, if the dispersal of species across different patches of habitats in a landscape is suppressed, localized extinctions cannot be compensated for by immigration from nearby habitat patches [87]. From a network perspective, this can be understood as species dispersal connecting (linking) certain habitat patches (nodes), and a governance implication resulting from these ecological interdependencies should be that the habitat patches ought to be managed as a *system* and not as isolated components in the landscape. Horizontal fit thus implies that the actors governing the habitat patches are collaborating in ways that increase their joint ability to manage (uphold) the connectivity of the patches. Concretely, this implies that two actors governing two habitat patches that are ecologically interdependent should coordinate their respective management activities in ways that preserve the integrity of this interdependency. Further, this ability would increase, all else being equal, if they collaborate. Hence, ideally, ecological links should be matched with social links (horizontal alignment captured by Fig. 3B, whereas Fig. 3A captures horizontal misalignment). Another example of horizontal fit derives from a situation in which two actors are managing the same habitat patch, which constitutes a prototypical example of the CPR problem. In this case, the potential benefit of collaboration is even higher, since this configuration implies resource competition (or in the absence of competitive use of the patch, there are still good reasons for the actors to coordinate their management activities, as is the case when they jointly engage in the maintenance of irrigation facilities). In such settings, extensive research has shown that if actors are collaborating in devising common rules that regulate resource use, this potentially competitive configuration does not have to lead to resource overexploitation [21,88,89]. It follows that if the actors have established a collaborative relationship (horizontal social link, Fig. 3D), they are better positioned to sustainably manage their common habitat patch than if they had not established such a relationship (Fig. 3C). Further, even though we here illustrate horizontal fit based on habitat connectivity in a landscape context, exactly the same reasoning applies to other settings where the ecological components and interdependencies are of a different sort. Furthermore, the same reasoning also applies when elaborating policy issues and their interdependencies.

Vertical fit turns our attention towards the links that connect actors with ecological components of interest (or policy issues). In short, if an actor only connects with a fraction of a system of interdependent habitat patches in a landscape, this represents vertical misfit since any secondary effects of certain land use practices emerging on interconnected patches would not directly affect the actor in question [54] (Fig. 3E). If the actor instead is connected to all habitat patches, which themselves are ecologically interconnected, the vertical fit would be at its peak (Fig. 3F). Naturally, in a landscape with many habitat patches, no single actor can, in most situations, govern all patches. Hence, there is an interplay between horizontal and vertical fit. If actors who are governing separate but interdependent patches are also collaborating (horizontal fit, Fig. 3B), the potential consequences of vertical misalignment could be reduced or diminished [83]. Further, although some level of vertical misalignment is typically to be expected in a large landscape, an analysis based on a multilevel ERGM can assess if and to what extent such misalignment is present. In other words, the analysis could reveal if there is positive or negative tendency among all actors to form social-ecological building blocks capturing vertical (or horizontal) alignment or misalignment (Fig. 3). As when elaborating horizontal fit above, the same reasoning applies in a broad range of environmental governance contexts, as it does when investigating policy issue interdependencies.

### Adaptive capacity from a social-ecological network perspective

**Figure 4.**
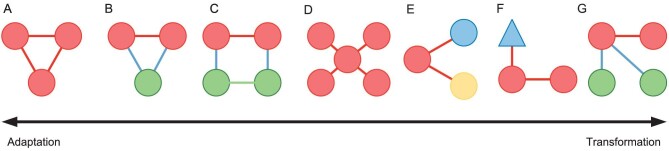
Social-ecological building blocks and their relationships to an adaptation versus transformation gradient. Red circular nodes represent (any type of) actors, and the blue and yellow circular nodes represent actors of different types, respectively. The blue triangular node represents an actor at a higher administrative level. The green circular nodes represent ecological components. The building blocks A–F are placed in accordance with their relationships to an adaptation versus transformation gradient (bottom arrow). Adapted from [95] in accordance with the CC BY 4.0 license (https://creativecommons.org/licenses/by/4.0/).

Social-ecological or institutional fit is an overarching perspective focusing on structural social-ecological alignment without detailing exactly if and how a good fit relates to specific processes and mechanisms that contribute to concrete management and governance activities that presumably favour the emergence of more desirable and sustainable social and ecological outcomes. For example, when two actors governing separate but ecologically interdependent habitat patches in a landscape collaborate (Fig. 3B), it represents horizontal social-ecological alignment that is theorized to contribute to more sustainable landscape governance. But the alignment does not explicate exactly how this could be accomplished, more that it is favourable that the actors in question collaborate. As stated, collaboration is a multidimensional concept covering interactions such as information exchange, coordination to maximize desired and agreed-upon objectives, and cooperation to negotiate e.g. trade-offs. Further, these different dimensions of collaboration impact different capacities that are affecting the ability to take collective action in different ways. Examples of such capacities are trust building [90], social learning [59], development of a shared vision [91], mutual acknowledgement of different viewpoints [35] and conflict resolution [92]. All of these are of importance in governing fast-changing landscapes, but one capacity that might be of relatively higher importance is adaptability. When a landscape changes rapidly and constantly, and therefore so too does the very basis for the livelihoods of the people residing in the landscape, adaptability with regard to land use and land use practices is of paramount importance. The importance of adaptability naturally also extends to many other environmental and governance contexts. Consequently, studies of adaptive capacity across various contexts have long attracted significant scholarly interest [14,23,93,94]. Studies focusing specifically on adaptive capacity using a SEN perspective are, however, still rare. A relatively recent contribution by Barnes and colleagues [95] takes an integrated perspective of SEN and institutional fit, and thus represents one of the few attempts so far. The study distinguishes between adaptive and transformative capacity, where the latter goes beyond adapting to changing conditions and instead relates to individuals’ and communities’ ability to fundamentally change their livelihoods and/or practices. Further, the study goes on to propose a number of social and social-ecological building blocks that are hypothesized to favour the respective capacities (Fig. 4). Of particular interest is that it is hypothesized that the social-ecological configurations outlined earlier, which relate to horizontal social-ecological fit, contribute to adaptive capacity (Fig. 3B and D).

This seminal study has spurred more research that has started to test these theories empirically. The importance of bonding and communicative social relationships among fellow fishers (Fig. 4A) for adaptation has been demonstrated in a study of Galician fishery communities in Spain, although it was also shown that social relationships based primarily on mutual trust can actually prevent fishers from adapting [96]. The mechanism behind the latter is not clear, but it is suggested that, for example, trust-based relationships can stimulate ‘too much’ social cohesion that might lock the actors into a status quo. In summary, the study lends some support to the importance of social transitivity (Fig. 4A), whilst also pointing out that this configuration might hamper adaptability if the relations are built too much on mutual trust. A study of islanders in Papua New Guinea provides results that in part contradict the previous ideas [97]. The configuration of two collaborating actors who each manage one of two interdependent ecological components (Fig. 4C) was actually negatively related to individuals’ adaptive capacity. Adaptability was here operationalized, among other things, as the willingness and capacity to build sea walls. It is clearly of importance to consider if and how this operationalization relates to adaptability in other contexts.

The studies discussed above focused on communities’ ability to adapt, taking into account their existing patterns of social-ecological relationships. A related question is if and how these relationships might change as a community goes through a period of great social and ecological change. Hence, the variable to explain is no longer adaptive capacity, but instead focus is set on if and how the network of social-ecological relationships changes as the community adapts to social and ecological change. A reef-fishing community in Papua New Guinea has been the focus for such a study [98]. Between 2002 and 2018, the coral cover decreased from 41% to 12%. As a result, fish stocks on the coral reefs are considered severely depleted. In the same time frame, the size of the human population on the island increased by 50%. These large changes have pressured the community to adapt. The focus of the study is, however, not to assess and evaluate how and to what extent they have adapted their fishing practices or livelihoods, but to investigate how they have changed their social-ecological relationships during the period 2016 to 2018. First, from a solely social network perspective, the social and ecological changes are aligned with an increased tendency of the islanders to tighten their social relationships. The tendency to form dense substructures (triadic closure, Fig. 4A) has increased, as has the islanders’ tendency to engage in relationships with others who are similar to themselves (homophily). The increased tendency for triadic closure aligns with theories about what structures are conducive for adaptability as outlined earlier, thus suggesting that islanders have increasingly engaged in triadic closure as a means of enhancing their adaptive capacity [95]. Further, during the years between 2016 and 2018, new leaders have emerged, who have also been forming many relationships with the islanders. This finding aligns with the earlier proposition that centralized social network structures would favour both adaptive and transformative capacities (Fig. 4D). Of the two horizontally aligned social-ecological building blocks that were proposed to favour adaptability (Fig. 4B and C), only the building block in which two actors connected to two interdependent ecological components collaborate was found to be positively associated with the ecological and social changes (Fig. 4C).

All in all, these few empirical studies provide mixed support for the previous propositions [95]. Although variability across cases is to be expected, these mixed findings indicate further research is needed to examine the general validity of the propositions across different contexts. Further, they also demonstrate the need to examine adaptive capacity and actors’ responses to changes, in concert, in order to better distinguish causes from effects. In particular, more work is needed in defining and operationalizing adaptive capacity in ways that are applicable and comparable across different contexts. The reviewed studies all focused on adaption to changes that strongly deviated from ‘everyday’ changes (e.g. continual but steady variability in the environment). In some contexts, the level of variability is relatively low, and the actors are more able to fine tune their practices given that the likelihood that conditions next year are going to look quite similar to this year is high. In these contexts, changes might be perceived and acted upon in different ways compared to contexts in which the naturally occurring level of variability is relatively high. When variability is high, adaptability is likely more ingrained in the everyday practices of how the environment is being governed. A reasonable assumption would thus be that actors have organized themselves differently in contexts with high variability in comparison to more stable contexts. In fast-changing landscapes where the ‘natural’ variability is high, adaptability would be a critical component of well-functioning environmental governance [19,99]. Thus, although we firmly acknowledge that small-scale fishers, such as the ones that were the focus of previous studies of large changes, are continually confronted by ‘naturally’ varying conditions, it nonetheless raises the question of whether the highly variable conditions in fast-changing landscapes imply that actors in these contexts are forming relationships with each other and the environment in ways that differ from relatively more stable environments where the everyday variability is lower. Again, we wish to emphasize that the conditions in the studied cases, before the induced large changes, were likely not overly stable—although they might not have been as variable as in fast-changing landscapes. In any case, more studies are needed to provide answers to these pressing questions. Unfortunately, however, adaptive capacity in fast-changing landscapes has, to the best of our knowledge, not yet been studied from a social-ecological-fit perspective using a SEN conceptualization. Thus, with the objective to *illustrate* the type of network-centric studies that we argue would be instrumental to furthering the understanding of the environment governance challenges of fast-changing landscapes, we present an empirical study of how rangeland managers (herders) form relations with each other and elements of the environment in a pastoral village of China where high variability is the norm.

## AN ILLUSTRATION OF THE CHALLENGES OF ADAPTIVE AND COLLABORATIVE ENVIRONMENTAL GOVERNANCE IN A CHANGING LANDSCAPE

This study is presented to illustrate how network thinking can be operationalized to improve our understanding of how rangeland managers address some important environmental and socioeconomic challenges in a pastoral village of Qinghai Province, China, on the QTP (Fig. 1). Specifically, the objective is to investigate if and how rangeland managers collaborate, while in parallel examine how they use and possibly relocate their herds among the available grazing areas from year to year. This landscape is subject to large intra- and inter-annual variabilities in precipitation and temperature, while also being confronted with stresses and shocks from rapidly changing institutions and policies, a fluctuating market, labour outflow and aging and competing land uses. Thus, the importance of adaptive capacity among the managers to sustain their livelihoods is high. To cope with the highly variable conditions, rangeland managers have developed and frequently taken a range of adaptive measures, such as reorganizing livestock production and forming marketing cooperatives, reallocating rangelands through leasing and merging, diversifying forage supply by planting artificial grasses and outsourcing, improving animal breeds, and purchasing livestock insurance. Reallocation occurs, for instance, when (i) managers form a cooperative and combine their patches to deal with the high variability in resource and labour availability, (ii) cooperatives and/or individual managers rent rangeland from other managers to enlarge operation scale or to use it for reserves or artificial grass fields, or (iii) managers lease out rangeland due to a labour shortage. For further details about the study area, data collection and analytical procedure, see Supplementary Data.

Our investigations follow the sequence in the previous section, i.e. we first focus on the social networks and identify some drivers behind the formation of social relationships among managers. Managers are furthermore hypothesized to utilize their social relationships when reallocating their livestock to different grazing rangeland patches through leases and other arrangements. Thus, we continue with assessing the institutional fit between the social networks and the pattern in which the managers utilize the system of grazing patches that largely characterizes this landscape, and that provides fodder for livestock (mainly sheep and yak). Finally, we investigate whether some of the social-ecological building blocks associated with fit coincide with the managers’ varying degrees of adaptability. Rangeland reallocation is, as stated, an important and frequent adaptation measure made by managers to cope with the highly variable conditions. In the study area, ∼70% of the managers changed the number of grazing patches they had during the study period 2020 to 2022. Therefore, we assess each manager's adaptability based on if and to what extent the manager has reallocated their livestock to different grazing patches during the study period. Considering that rangeland reallocation is such a common adaptation measure, high adaptability is here assessed based on whether the managers have changed the number of grazing patches they utilize between 2020 and 2022 markedly more than most other managers. By applying a threshold of at least a 50% change in the number of utilized grazing patches during the study period, 34% of all the managers were assessed as highly adaptive. Although the threshold of 50% is a bit arbitrary, it was chosen to single out a reasonably sized proportion of the managers that were more adaptive than others (a sensitivity analysis showed that the significant results presented further down remained when modifying the threshold, see Supplementary Data).

**Figure 5.**
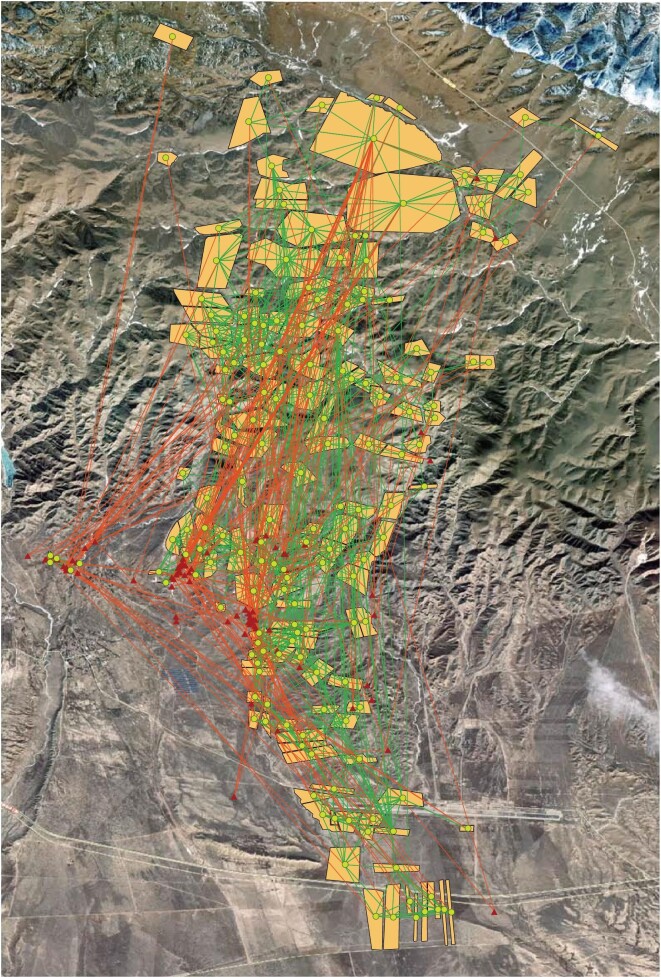
A rangeland system in Qinghai, China, described as a social-ecological network. Red triangles represent social actors—rangeland manager (herder) households, and their positions correspond to the herders’ homestead locations. Green points represent ecological nodes—rangeland plots/patches (yellow polygons), and their positions correspond to the geometric central points of the polygons. Green lines illustrate the ecological linkages between rangeland patches, based on assumed regular species dispersal in the landscape; red lines capture the management relationship defining which rangeland patches the managers use for livestock grazing. For clarity, the social linkages between managers are not shown. See Supplementary Data for further information.

The starting point for the analysis is to construct a SEN consisting of the rangeland managers, their social relationships, their use of grazing patches, the grazing patches themselves and finally the links between the patches that were assessed based on geographical distances (patches that were within 1000 m of each other were considered connected, see Supplementary Data) (Fig. 5).

Two types of social relationships were assessed: affection (i.e. if they perceived others as trustworthy, reliable and/or friendly) and collaboration (if they worked together within the frames of certain organized activities and/or organizations). We do not distinguish these relationships in terms of their potential contribution to learning, cooperation and coordination, but we assert the affection network to be less constrained by norms and practices than the organizational network since there are likely expectations within the latter that, for example, a manager should engage in collaborative relationships with certain others. Furthermore, information about the managers’ group affiliation and their social status were collected (see Supplementary Data). Then, we conducted statistical network analyses based on the ERGM and autologistic actor attribute models (ALAAM, see Supplementary Data) using the software MPNet [53], focusing on certain social-ecological building blocks.

Both social networks encompass transitivity and homophily (Table 1, see also, Supplementary Data). Both of these tendencies are common across the two types of social networks, but what specific attributes give rise to homophily typically varies between contexts. In this case, being part of the same geographically and kinship-defined group (managers close in blood lineage usually cluster geographically) coincides with tendencies to form social relationships (Table 1). Even though comparisons across different networks are a daunting task when using the ERGM [100], we nonetheless note that the homophily effect is strongest in the affection-based social network. Furthermore, transitivity has, as outlined earlier, been associated with adaptability (Fig. 4A). Both the affective and the collaborative social networks can thus be asserted as favouring adaptability, albeit this assertion is only based on considering this specific building block.

**Table 1.** Results from ERGM applied to the SENs of the rangeland managers.

	Affection-based social network		Organization-based collaborative social network	
Effects^a^	Parameter	Standard error	Parameter	Standard error
Density of links (EdgeA)	-5.4365*	0.286	-4.7069*	0.173
Transitivity (ATA)	0.4636*	0.103	0.4607*	0.104
Number of links related to high social status (social.status_ActivityA)	0.2703	0.192	1.0271*	0.208
Homophily of actors with high social status (social.status_InteractionA)	-0.3596	0.901	0.0735	0.443
Homophily of actors residing in the same area within the village (Group_MatchA)	2.8095*	0.317	1.533*	0.192
Two socially connected actors sharing a grazing patch (TriangleXAX) (Fig. 3D)	0.1895*	0.079	0.2348*	0.084

^a^The configurations (building block) used in MPNet within parenthesis. *Significant at 0.05 level.

The most notable difference between the two social networks is, however, the role of social status. High social status here refers to holding positions as government officials, village leaders and/or enterprise or cooperative managers. Social status is a strongly significant driver of relationships in the collaborative network, but not in the affective network (Table 1). This indicates that managers might engage in collaboration with high-status individuals to, for example, get access to various types of resources (material and immaterial); but it may also suggest there are norms and practices that motivate them to form such relationships, especially in organizational settings.

One characteristic of possessing high social status, given the way it is assessed in this study, is the rangeland managers’ increased likelihood of having established social relationships with other actors operating at higher administrative levels (cf. linking social capital [101]). Hence, since high-social-status managers tend to have more collaborative relationships than others, this would thus coincide with the building block previously assessed as important mostly for transformative capacity [95] (Fig. 4F). Leadership executed by such potential boundary spanners has been shown to help amass support in attempts to address environmental problems through transformational changes [28,29]. In the study area, this could be potentially exemplified by promoting the transformation of animal husbandry from a scattered, natural grazing pattern to an intensive, centralized breeding pattern. In fact, studies have shown that local elites in similar contexts have mobilized their personal contacts to obtain financial and technical support for the whole community to initiate such transformational changes [102,103].

The social-ecological fit is quite similar across the two social networks (Table 1, see triangleXAX). Only one of the building blocks commonly associated with fit was significant, namely the fully connected inverted social-ecological triangle (two managers that are sharing a grazing patch are interacting socially, Fig. 3D). This suggests that managers that utilize common rangelands use their social relationship to coordinate their grazing activities to avoid overexploitation and/or build their relationship when combining and/or sharing their grazing patches. Further, this building block is also more associated with adaptive rather than transformative capacity (Fig. 4B).

We then turn our attention to the individual managers who were highly adaptive during the studied time frame. The ALAAM modelling did not allow us to include all building blocks of interest due to model convergence issues, thus we had to resort to a rather simplistic model and instead rely on goodness-of-fit estimations (Table 2, Supplementary Data). These assessments are not as statistically robust as when the building blocks of interest are directly included in the models (i.e. their parameter values are estimated). Nonetheless, goodness-of-fit estimations can give strong indications about whether certain building blocks are markedly over- or underrepresented in the empirical network. The results indicate that if two herders are socially connected and share a grazing patch, it is more likely than chance that both of them are highly adaptive. In other words, a fully connected inverted social-ecological triangle (Fig. 3D) makes it more likely that both managers are highly adaptive. It is important to point out that the same building blocks do not make it more likely that one of the managers is highly adaptive, regardless of whether the other is highly adaptive or not. Hence, there seems to exist interaction effects implying that managers that need to markedly change the number of grazing patches use their social relationships to seek out others that also need to adapt strongly, and that both of them, in that process, will share at least one grazing patch. Since rangeland reallocation is inherent in the managers’ routine adaptive practices, we assert that the fully connected inverted social-ecological triangle is more associated with adaptive rather than transformative capacity. These effects were, however, only seen for affective social relationships and not for collaborative relationships. One possible conclusion would thus be that affective social relationships are, in this context, more important for the managers’ adaptive capacities than their collaborative relationships.

**Table 2.** Goodness-of-fit drawn from the ALAAM analysing high adaptability of rangeland managers.

Effect^a^	Frequency in empirical network	Estimated frequency	Standard error on estimated frequency	T-ratio
Affection-based social network				
Two socially connected and highly adaptive managers are sharing a grazing patch (TXAX-2A)	19	6.175	3.993	3.212*
Organization-based collaborative social network				
Two socially connected and highly adaptive managers are sharing a grazing patch (TXAX-2A)	5	3.835	2.592	0.449

^a^The configurations (building blocks) used in MPNet are within parentheses. The estimated (log) propensity of link to grazing patches from highly adaptive managers (ActivityXA) was −0.3401 and the estimated (log) frequency of highly adaptive managers (DensityXA) was 0.4424. These estimations together, in combination, control for the tendency for highly adaptive managers to have more or less links to grazing patches, and the sheer number of managers that were highly adaptive, respectively. The values in the table are drawn from goodness-of-fit estimations, where the estimated frequencies of TXAX-2A are compared with the frequencies in the empirical networks. If the observed frequencies deviate more than 2 standard errors from the estimated frequencies, the effect is deemed statistically significant at the level of 0.05 (marked with *, cf. [72]). No other configurations in the goodness-of-fit estimations were significant (see Supplementary Data).

Although these results derive from a single case study and should thus be considered with caution, we nonetheless assert that our results from this highly variable landscape align with the previous proposition (Fig. 4B) stating that when two actors sharing a common ecological component are socially connected, their adaptive capacity is enhanced. But only if the social relationship is established voluntarily and encompasses trust, reliability and/or friendship. Further, the results also suggest that adaptability in highly variable contexts is at least in part a social phenomenon, meaning that actors develop their adaptive capacity jointly and in concert with others they trust.

In summary, we argue that these analyses and results demonstrate the applicability of using a network perspective in furthering our understanding of how actors act and operate in fast-changing landscapes where the interdependencies between societies and ecosystems are strong and have direct and significant consequences for both livelihoods and landscapes. Such understanding is imperative in order to devise new policies, practices and governance approaches that are better able to address current and forthcoming challenges to these tightly integrated SESs.

## SUMMARY AND FUTURE RESEARCH OPPORTUNITIES

The network perspective has contributed substantially to the understanding of human–nature interactions across different contexts by adopting an interdisciplinary social-ecological analytical frame. While this stream of research is still at an early phase, multiple case studies have been conducted across the world, and more are on the way. These developments have been acknowledged and emphasized through a series of recent reviews [55,56,83], and some of the key insights derived from these studies are also presented here. So far, however, studies applying this research perspective in inherently dynamic landscapes that are also undergoing large-scale changes are still virtually absent. The case study presented here is included as an illustration to showcase how the SEN-centric perspective can help to address some of the questions pertaining to how actors in a rapidly changing landscape choose to engage in collaboration with others, and if and how these relationships affect how they use the land, now and in the future. Besides the fact that more work is clearly needed to more thoroughly ascertain if and to what extent these case-based insights are more generally applicable, we wish to highlight some issues that we argue are of key importance in order to move this new research frontier forward.

### An explicit dynamic perspective

Reality is not static, but changes constantly. The natural environment is constantly changing, as do actors and institutions as their preferences change, new knowledge is developed, new constellations of power and influence emerge, and the natural environment changes—thereby closing a feedback loop between societies and ecosystems [16,29,104]. Thus, while acknowledging the immense value of cross-sectional studies and the hurdles in gathering and analysing longitudinal data, we nonetheless argue that more work should be devoted to empirical studies that investigate different phenomena over time. This is clearly paramount in developing a better understanding of highly dynamic SESs, such as fast-changing landscapes [3]. Studying phenomena over time also provides means to empirically and analytically differentiate causation from correlation [83,105]. Ideally, we want to be able to address the ‘why’ questions, since such knowledge is crucial to devising measures that could affect the current state of affairs in desirable directions (acknowledging that agreeing on what is a desirable direction is far from trivial).

### Contextual sensitivity

That context matters has long since been established (e.g. [92]). But it does not necessarily mean there are no generalizable insights to be drawn from empirical studies in different contexts. Even though much of this article is devoted to fast-changing landscapes, many insights from other contexts are also applicable to this particular context, and vice versa. Further, while different fast-changing landscapes all have their own unique characteristics, there might be insights to be drawn that are applicable across landscapes. Thus, an ever-present challenge is if and how to develop generalizable insights while taking contextual differences into account [106]. Not only are multiple studies across different contexts needed for such an endeavour, so are empirically, theoretically and methodologically informed analytical approaches [105,107]. We argue that the network perspective could be an important tool in such a toolbox since it provides the means by which to conceptualize and operationalize patterns of human–nature interactions in ways that could be comparable across different contexts.

### Conflict and collaboration

Much of the social and SEN-centric research is concerned with positive relationships between actors [33]. By positive we mean relationships that are (potentially) beneficial to the actors in the sense that the relationship can provide them with access to material and immaterial resources of different kinds (e.g. knowledge, information, support, funding and environmental resources) that could be of value either to themselves directly and/or through enabling collective action that could support actors in addressing issues they cannot address themselves. Many relationships are, however, not positive in that sense. For example, disaffection, competition and distrust are types of relationship deemed as negative [108]. Many if not most environmental problems involve different groups of actors with different interests having both positive and negative relationships with each other, sometimes even with the very same actors (e.g. [109]). Thus, there are interaction effects between positive and negative relationships, emphasizing that a joint ability to address common problems is likely affected by certain combinations of positive and negative relationships (or in other words, by combinations of collaborative and conflictual relationships). Further, negative relationships could even be critical in instigating changes because they prompt actors to act, although they could also be effective in suppressing action, and/or they could direct action in ways that aggravate an environmental problem [35]. Hence, there is a need to better understand if and how positive and negative relationships interact, and furthermore, how interaction effects might be driven by how the actors are utilizing different environmental resources. Furthermore, what social and ecological consequences derive from such interactions? Here, the network perspective could be instrumental in exploring these largely uncharted territories [33].

## DATA AVAILABILITY

The data are available upon request.

## Supplementary Material

nwad019_Supplemental_File
