# Supplementary material for: A network perspective of human–nature interactions in dynamic and fast-changing landscapes
Source: Natl Sci Rev. 2023 Jan 17;10(7):nwad019. doi: 10.1093/nsr/nwad019 (PMC10232045; doi:10.1093/nsr/nwad019)
Supplement: nwad019_Supplemental_File [file nwad019_supplemental_file.pdf]

A network perspective of human-nature interactions in dynamic and fast-changing landscapes

Örjan Bodin and Haibin Chen

SUPPLEMENTARY DATA

Study area

Qinghai Province of China is situated on the Tibetan plateau (31°36′–39°19′N, 89°35′–103°04′E), with an average altitude of more than 3000 m. Due to the high altitude, the climate in Qinghai is characterized by hypoxia, frigidity and aridity, with a mean annual precipitation of 50–550 mm and a mean annual temperature of −5–8.5°C. Due to a diverse and complex terrain, the climate also features large variation in space and time, resulting in significant spatiotemporal unevenness in biomass productivity. As of 2021, the province had a human population of 5.94 million, with a high proportion of ethnic minorities, mainly Tibetans. It has an abundant alpine rangeland resource, with a total area of approximately 41.93 million ha, and livestock production is the main livelihood source for a majority of rural residents. The main reared livestock species are Tibetan sheep and yak, both adapting well to the hypoxia and cold climate. However, due to rapid population growth and increasingly intensified landscape alteration from residence construction, industrial development and livestock grazing, rangeland degradation and even desertification are prevalent across the landscape. Furthermore, with the acceleration of globalization, urbanization and climate change, pastoral villages in Qinghai also have to be confronted with ongoing shocks and stresses from labor outflow, frequent droughts and snowstorms, and market volatility.

Since 1980s, the Household Contract Responsibility System has been gradually rolled out across the pastoral Qinghai. Under this new rangeland tenure system, the original village-owned rangelands and livestock were contracted out and allocated to individual households, in the hope of better addressing the challenges of common pool resource management. The original cooperative production pattern has since dismantled, and herdsmen (rangeland managers) have now become atomic actors and have to independently face all the external risks, notably from the climate hazards and market competition, resulting in an increase in their livelihood vulnerability. The division of rangeland not only results in rangeland fragmentation and increases livestock production cost, but also breaks the integrity of natural landscape. It also greatly reduces the mobility of herds. Long-range and large-scale nomadism is now impossible, and animals can only graze on household contracted rangelands. The dwindling mobile space intensifies the trampling of livestock on rangeland and causes the problem of “hoof disaster”, weakening the ecosystem resilience. In addition, to curb the rangeland degradation trend, several Payment for Ecosystem Services programs have been successively rolled out in Qinghai since the early 2000s, e.g., the Returning Grazing Land to Grassland project, and the Rangeland Ecological Protection Subsidy and Reward scheme. The main measures taken include year-round or seasonal grazing cessation, rotational

grazing and forage-livestock balance, as overgrazing is deemed as the primary cause to rangeland degradation. Consequentially, the traditional natural grazing practice has been restrained to a certain extent. To maintain herders' livelihood, confined livestock feeding has since been encouraged as an alternative to the open grazing practice, in parallel with the implementation of the herdsman settlement initiatives.

It can be seen that herders in Qinghai have been confronted with a dynamic and fast-changing landscape, and have to constantly adapt to a multiplicity of rapid changes and shocks from climate, economy, demography, and changing policies. Despite all these challenges, hundreds of years of nomadic practice has nonetheless developed an entrenched culture of reciprocity and trust in the pastoral villages. Collaboration between herders is indeed not uncommon, although it may take on a relatively scattered, spontaneous fashion [1]. During busy farming season, hand lending is a common way to cope with labor shortage. Various forms of cooperatives have also been established to facilitate production cooperation and enhance bargaining power in the market. Families may pool their rangelands together and allow the access of each other's animals, to adapt to the spatial and temporal variation of local biomass productivity. A few grazing patches remain community-owned and are kept as public reserves, and can be used by all villagers in case of emergencies. Renting rangeland from peers is also a common practice to offset the impacts of grazing restriction policy. Moreover, to mitigate the problem of forage shortage, some herders have started planting artificial grasses and/or outsourcing forage. To control the risk of livestock death due to climate hazards such as drought and snowstorm, herders are now proactively breeding improved animal varieties and/or purchasing livestock insurance.

Data gathering and formatting

Two rounds of questionnaire surveys were administered to collect the data. Yangrang village, situated at Gonghe County in Hainan Tibetan Autonomous Prefecture of Qinghai, was selected as the survey object. The village is typical for the study area given the population is mainly made up of Tibetans and livestock production is the main income source for most residents. The first survey was carried out between July and August of 2020. Questions cover the demographic characteristics of herder households (group affiliation, family size, labor size, social status, annual income), attributes of household heads (gender, age, education, Mandarin proficiency), rangeland operation (contracted land acreage, rented and leased land area, number of grazing plot and grazing area in use), herd size, as well as their social networks [1]. In this study, only group affiliation, social status, number of grazing plot, and social networks were analyzed. To build networks, the full-sample survey method was applied, that is, all households in the village were surveyed (if they were available). Meanwhile, the roster method was used to help the respondents to identify their social relationships, which is believed to be superior to the recall method in capturing in particular weaker social ties. Before the survey, a village leader helped to compile a name list of all herder households in the village. From that list, the respondents were asked to select up to seven households that they recognized as having social relationships with. Four types of social relationships were gathered, i.e., affective relations, collaboration in organizations, information exchange and resource exchange. Only the first two relationships were used in this study (Table A1). The respondent could indicate the strength of their social relationship on a scale of 1–5, where 1 denotes the weakest and 5 the strongest. For this

study, all relationships with a strength > 0 were included. The respondents were permitted to add new herders to the list, but to limit any biases, these were not included in the analysis.

Another round of survey was run in July of 2022. Questions as regards to rangeland operation were asked again (number of grazing plots and grazing areas in use), to trace households' land use change during these two years. In addition, to establish herder-rangeland linkages, we also asked each respondent to identify the location and boundary of their lands in use. Finally, a total of 99 households were interviewed. After removing those with missing data or obvious errors, 85 valid households were retained, by which 199 plots of rangeland patches were used.

With the collected data at hand, two social networks were built (based on the 2020 survey), illustrating herder households as nodes and their social relationships as links (Fig. A1). The 199 rangeland grazing plots were defined as ecological nodes. Social-ecological links were built based on the relationships between herders with land plots/patches, i.e. the land used for grazing in 2022. Ecological links between plots were built by applying the buffer zone analysis method, where 1 km buffer zones were drawn around the boundary of each land plot, and an ecological link between two land plots was established when one was located in the other's buffer zone. The use of 1 km edge-edge buffer zone is based on the following two considerations. First, 1 km corresponds approximately to the maximal distance of regular and frequent mobility of livestock, except for long-range seasonal migrations between summer and winter pastures. Second, the potential dispersal distance of rodents (*zokor*, *marmot* and *plateau pika*), which represents a prominent environmental problem in the study area given its severe negative impacts on human and ecological communities in terms of reducing primary productivity and increasing risk of zoonotic diseases dispersion. Addressing such a problem also requires substantial coordinated actions between neighboring herders. Hence, we assert that two land plots within a 1 km distance represent a *system* of grazing patches that ought to be managed in concert.

Table A1 Types of network links in the SENs

**Type**	**Link**	**Description**
Social	Affective relations	Kinships, friendships or other social relationships that are developed based on personal affections and perceived as trustworthy, reliable, or friendly.
	Organization-based collaboration	Collaborative relationship that are developed within the frames of certain organized activities or organizations, such as government bodies, village committees, cooperatives, enterprises, etc.
Social-ecological	Rangeland use	Management relationship between land plots/patches and herders.
Ecological	Species dispersal and livestock movement	Based on assumed regular movement of livestock and dispersal abilities of rodents in the landscape.

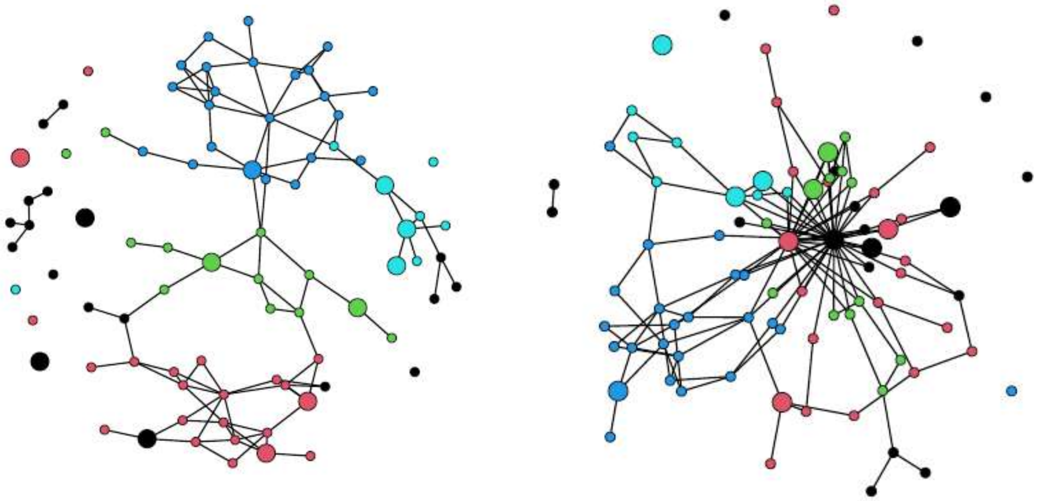

Fig. A1 *Social networks based on (left) affective and (right) organization-based collaborative relationships. The colors of the nodes represent group affiliation, and the larger nodes represent households with high social status.*

Analyses

We applied multilevel ERGM and ALAAM using the software MPNet [2]. ERGM is described extensively elsewhere [3], and is conceptually similar to regression analyses where the analysists specify a certain set of configurations (building blocks) as the independent variables, and the links in the observed network constitute the dependent variable. The estimated coefficients of the configurations signify if these configurations are significantly contributing to explaining the network, based on the conditional likelihood that links are formed to either increase or decrease the frequencies of these configurations in the network. ALAAM [4] are based on the same underlying statistical approach as in ERGM. But instead of treating the links in the observed network as the dependent variable, ALAAM treats a chosen attribute of the nodes as the dependent variable (which has to be binary for multilevel models, which meant we had to define a threshold for a herder being highly adaptive or not). Hence, ALAAM analyzes if the observed network structure, along with attributes different from the one chosen as the dependent variable, can explain the chosen nodal attribute.

ERGM and ALAAM uses Markov Chain Monte Carlo (MCMC) estimation procedure, which imply that a specified model does not always converge (the model cannot be adequately fitted to the data). In such cases, it could still be possible to estimate the effects of certain configurations that could not be included in the model, although such estimations are less statistically robust. Such estimation could be done by fitting a simpler ERGM model (less configurations) to the data that does converge. And then conduct a goodness-of-fit (GOF) test. A GOF is conducted by simulating a large number of networks, typically several thousand, based on the simpler model. Then, the frequencies of the configurations that could not be included in the model are compared across the observed network and the large sample of simulated networks. If the observed frequencies deviate more than two

standard errors from the simulated networks mean values (T-ratio > 2), the corresponding configurations can be considered as deviating significantly from what would be expected by chance (cf. [5]). The same approach can be used in ALAAM.

Results

We were able to fit ERGMs including most of the configurations of interest (for example, see Fig. 3 and Fig. 4 in the main text). Any remaining configuring not included in the models did not deviate more than two T-ratios from the sample means in the GOF. Hence, we assert our results from ERGM (Table 1 in the main text) adequately represent all configurations of interest for this particular study.

Further, we could not fit a ALAAM including the social-ecological configurations we deemed most important for this study, namely Star2-AX-EgoA (the more land plots a herder is using while also being connected to many other herders, the more likely it is highly adaptive), TXAX-1A (the more often a herder is situated in a social-ecological building block consisting of two socially connected herders sharing a land plot, the more likely the herder is highly adaptive), TXAX-2A (as TXAX-1A, but with the extension that both herders are highly adaptive), C4AXB-1A (the more often a herder is situated in a social-ecological building block consisting of two socially connected herders each using separate land plots that are interconnected, the more likely the herder is highly adaptive), C4AXB-2A (as C4AXB-1A, but with the extension that both herders are highly adaptive). TXAX- and C4AXB-represent the social-ecological building blocks D and B in Fig. 3 in the main text, respectively. Star2-AX-EgoA relates to configuration G in Fig. 4 by representing a herder that is using many land plots while also being socially connected to many other herders. The GOF revealed that only TXAX-2A deviated more than two T-ratios from the sample means, but for only one of the two types of social relationships being investigated in this study (see Table 2 in the main text). Thus we assert none of the other configurations deviate significantly from chance, and that TXAX-2A is the only one, from the configurations outlined above, that significantly contributes in explaining why a herder is highly adaptive.

A sensitivity analysis was also carried out where we changed the threshold for a herder being considered as highly adaptive to 40% and 60% change in the number of used grazing plots, respectively. The results for TXAX-2A remained significant with these modified thresholds. Further, we also tested for “simple” social contagion, i.e. if herders are more likely to be highly adaptive if they are socially connected to other highly adaptive herders. But no such effect was revealed as significant.

Questionnaire details

All questions were asked in Tibetan with the help of local translators, and the translated questions below have been slightly modified and abbreviated for clarity.

The following questions were used to assess group affiliation

- Which villager group does your household belong to? (please select one from number 1 to 5) (*groups were predefined based on geographical location*).
- The geographical location of your homestead: longitude_______ latitude_________ *(Investigators record GPS readings onsite)*.

The follow question was used to assess social status

- How many in your household are serving as government officials, village cadres, and/or enterprise executives? *(all households responding with a number > 0 were deemed as possessing high social status)*_

The following questions were used to assess land use

- How many grazing patches of rangeland are used by your household now? (*asked in 2020 and 2022*)
- Where are these patches situated? (*only asked in 2022, and the geographical location and boundary of each rangeland plot was identified by respondents and drawn on map by investigators).*

References

1. Ding, R, Shao, L, Chen, H. Curbing overstocking on rangeland through subsidies, rewards, and herders' social capital: Lessons from Qinghai province, China. *Journal of Rural Studies*. 2021; **87**: 361-74.
2. Wang, P, Robins, G, Pattison, P*, et al.* MPNet: Program for the simulation and estimation of (p*) exponential random graph models for multilevel networks. Melbourne, Australia; 2014.
3. Lusher, D, Koskinen, J, Robins, G. *Exponential random graph models for social networks: Theory, methods, and applications*. New York: Cambridge University Press; 2013.
4. Daraganova, G, Robins, GJErgmfsnT, methods, applications. Autologistic actor attribute models. In: Lusher, D, Koskinen, J, Robins, G (eds.). *Exponential random graph models for social networks: Theory, methods and applications*. New York: Cambridge University Press; 2013. 102-14.
5. McAllister, RRJ, Taylor, BM, Harman, BP. Partnership networks for urban development: how structure is shaped by risk. *Policy Studies Journal*. 2015; **43**(3): 379-98.
